# Prévalence et facteurs associés au portage des anticorps anti-VHC chez des femmes enceintes à Cotonou

**DOI:** 10.11604/pamj.2020.36.182.23122

**Published:** 2020-07-14

**Authors:** Moufalilou Aboubakar, Aboudou Raïmi Kpossou, Bignon Rosalie Gloria Hermione Glago, Amel Gildas Aguiah, Zafy Hairou Mboreha, Jean Sehonou

**Affiliations:** 1 Centre Hospitalier Universitaire de la Mère et de l´Enfant Lagune (CHU-MEL), Cotonou, Bénin,; 2 Clinique Universitaire d´Hépato-gastro-entérologie, Centre National Hospitalier et Universitaire Hubert Koutoukou Maga (CNHU-HKM), Cotonou, Bénin,; 3 Clinique Universitaire de Gynécologie-obstétrique, Centre National Hospitalier et Universitaire Hubert Koutoukou Maga (CNHU-HKM), Cotonou, Bénin

**Keywords:** Hépatite C, prévalence, facteurs associés, diabète gestationnel, femmes enceintes, Cotonou (Benin), Hepatitis C, prevalence, associated factors, gestational diabetes, pregnant women, Cotonou

## Abstract

**Introduction:**

l´hépatite C est une infection dont la transmission mère-enfant est possible. L´objectif de ce travail était d´étudier la prévalence du portage des anticorps anti-VHC chez des femmes enceintes à Cotonou et d´identifier les facteurs qui y sont associés.

**Méthodes:**

il s´est agi d´une étude transversale menée du 01/06/2018 au 01/09/2018 auprès de 253 gestantes reçues pour des soins prénatals dans quatre grandes maternités de Cotonou (Bénin). Les anticorps anti-VHC avaient été détectés par des tests rapides d´orientation diagnostique. Un échantillon de sang veineux avait été prélevé chez les gestantes avec anti-VHC positifs en vue de tests sérologiques de confirmation et d´un dépistage du diabète gestationnel.

**Résultats:**

la prévalence des anticorps anti-VHC était de 1,2% (3/253 gestantes). Les facteurs associés au portage du VHC n´avaient pu être identifiés compte tenu du faible nombre de cas positifs. Cependant, les gestantes porteuses des anticorps anti-VHC avaient une moyenne d´âge élevée (32 ± 3) comparativement au reste de la population (29,58 ± 5,5). Les facteurs de risque potentiels d´infection par le VHC retrouvés étaient les scarifications, le piercing, le tatouage, le partage du matériel de manucure, un antécédent d´intervention chirurgicale et de transfusion sanguine. La prévalence du diabète gestationnel dans notre étude était de 7,9% (20/253). Aucune association n´était trouvée entre le diabète gestationnel et l´hépatite C.

**Conclusion:**

la prévalence des anticorps anti-VHC chez les gestantes à Cotonou était faible. Une étude d´envergure nationale s´avère nécessaire afin d´identifier les facteurs associés à cette infection.

## Abstract (en)

**Introduction:**

hepatitis C is an infection which can be passed from mother to child. The purpose of this study was to investigate the prevalence of colonization by anti-HCV antibodies in pregnant women living in Cotonou and to identify factors associated with it.

**Methods:**

we conducted a cross-sectional study of 253 pregnant women admitted for prenatal care in four major maternity hospitals in Cotonou (Benin) from 01/06/2018 to 01/09/2018. Anti-HCV antibodies were detected using rapid diagnostic tests. A venous blood sample was collected from pregnant women tested positive for anti-HCV before confirmatory serological tests and screening tests for gestational diabetes.

**Results:**

the prevalence of anti-HCV antibodies was 1.2% (3/253 pregnant women). Factors associated with HCV carriage couldn’t be identified given the low number of positive cases. However, pregnant women who were carriers of hepatitis C antibodies had higher mean age (32 ± 3) compared to the remainder of the population (29.58 ± 5.5). Potential risk factors for HCV infection were scarifications, piercing, tattooing, sharing of manicure equipment, a history of surgery and blood transfusions. The prevalence of gestational diabetes in our study population was 7.9% (20/253). No association was found between gestational diabetes and hepatitis C.

**Conclusion:**

the prevalence of anti-HCV antibodies in pregnant women living in Cotonou was low. A national-level study is needed to identify factors associated with this infection.

## Introduction

L'infection par le virus de l´hépatite C (VHC) touche environ 71 millions de personnes de par le monde (soit 1% de la population mondiale), dont 75 à 85% développeront une hépatite chronique [1-3]. En Afrique subsaharienne, la prévalence des anticorps anti-VHC est estimée à 2,9% [4]. L´Egypte porte le taux de prévalence d´anticorps anti-VHC le plus élevé au monde, avec 15% en 2015 [5]. Le mode de contamination est principalement parentéral; toutefois la transmission verticale est possible avec un taux de 5 et 7% en cas d´infection par le VHC seul, et entre 15 et 18% en cas de co-infection VIH-VHC [6, 7]. Chez les femmes enceintes, la prévalence des anticorps anti-VHC serait similaire à celle dans la population générale (1-8%) avec des variations d´un pays à un autre [8].

Dans la pratique, le dépistage du VHC chez la femme enceinte n´est pas systématique contrairement à celui des infections à VIH et à VHB [9]. Actuellement il n´existe aucun protocole de prévention de transmission mère-enfant dans le cas de l´infection à VHC [3]. Depuis 2014, avec l´arrivée d´une nouvelle génération d´antiviraux d´action directe (AAD), le traitement de l´infection par le VHC donne de très bons résultats [10, 11]. Le diagnostic prénatal du VHC trouverait ainsi son intérêt. Au Bénin précisément à Tanguiéta, De Paschale *et al*. avaient trouvé en 2014 une prévalence de 7,4% de portage des anticorps anti-VHC chez les gestantes [12]. Il nous a paru important de mener cette étude afin de déterminer la prévalence du portage des anticorps anti-VHC et d´identifier les facteurs qui y sont associés chez des femmes enceintes à Cotonou, la plus grande ville du Bénin.

## Méthodes

Il s´est agi d´une étude transversale, à visée descriptive menée sur une période de trois mois allant du 01/06/2018 au 01/ 09/2018. Elle s´est déroulée à Cotonou dans quatre centres dont trois grands centres hospitaliers universitaires et une clinique privée de gynécologie obstétrique: la Clinique Universitaire de Gynécologie Obstétrique (CUGO) du Centre National Hospitalier et Universitaire Hubert Koutoukou Maga (CNHU-HKM), le service Mère du Centre Hospitalier et Universitaire de la Mère et de l´Enfant Lagune (CHU-MEL), la maternité de l´Hôpital d´Instructions des Armées-Centre Hospitalier Universitaire (HIA-CHU) et la clinique privée Point E de Cotonou. Ont été incluses toutes les gestantes régulièrement suivies en consultation prénatale dans les dits centres et ayant donné leur consentement pour participer à l´étude.

Chaque gestante a bénéficié d´une séance de counselling sur l´infection à VHC (modes de transmission, l´existence d´un traitement efficace...). Les gestantes consentantes ont bénéficié d´un test rapide d´orientation diagnostique (TROD): InTeC^®^HCV Rapid Test (InTeC Products Inc; Chine). En cas de TROD positif, un test sérologique ELISA (Enzyme-Linked Immunosorbent Assay) de confirmation était demandé et la gestante adressée en consultation d´hépato-gastroentérologie. Dans notre étude, le diabète gestationnel a été défini selon les critères de l´*International Association of Diabetes Pregnancy Study Group* (IADPSG). Il s´agit de la présence d´un des critères suivants: une glycémie à jeun supérieure ou égale à 0,92g/L, une glycémie à une heure supérieure ou égale à 1,80g/L ou une glycémie à deux heures supérieure ou égale à 1,53g/L après l´épreuve d´hyperglycémie provoquée par voie orale réalisée après une charge orale de 75g de glucose. Les variables étudiées ont porté sur les données socio-démographiques, les facteurs de risque, les examens paracliniques. Les données avaient été saisies et analysées à l´aide du logiciel SPSS Statistics dans sa version 20.0.

## Résultats

**Caractéristiques de la population étudiée:** au total 253 gestantes ont été recrutées dans l´étude. La moyenne d´âge était de 29,58 ±5,5 ans, avec des extrêmes de 14 ans et de 45 ans. La tranche d´âge la plus représentée était celle de 20 à 39 ans (92%; n=233). La majorité était de religion chrétienne (75,9%; n=192) et 62,4% (n=158) étaient mariées. L´hypertension artérielle (HTA) était l´antécédent médical le plus fréquent (14%; n=36) et 47,4% (n=120) avaient bénéficié d´une intervention chirurgicale. Les patientes ayant un antécédent de transfusion sanguine représentaient 10,3% (n=26). Parmi les gestantes, 12% (n=31) étaient séropositives au VHB et 1,7% (n=5) séropositives au VIH. Une grande proportion des gestantes ne connaissait pas son statut sérologique antérieur au VHC (82,2%; n=208). Une seule se connaissait porteuse du VHC.

**Prévalence des anticorps anti-VHC chez les gestantes:** dans l´étude, la proportion de gestantes positives aux anticorps anti-VHC par TROD était de 1,2% (3/253 gestantes). Ces résultats ont été tous confirmés par des tests ELISA. Deux gestantes étaient au 2^e^ trimestre et une au 3^e^ trimestre de grossesse.

**Facteurs de risque de portage des anticorps anti-VHC retrouvés chez les gestantes** (Tableau 1): les principaux facteurs de risque retrouvés chez les gestantes porteuses des anticorps anti-VHC étaient la présence de scarifications (2 gestantes/3), un antécédent d´intervention chirurgicale (2 gestantes/3) et le partage de matériel de manucure (2 gestantes/3). Il n´était pas retrouvé de notion d´hépatite C dans l´entourage de ces gestantes. Le statut du partenaire n´était connu que dans un cas et il était séronégatif au VHC.

**Tableau 1:** facteurs de risque de portage des anticorps anti-VHC au sein des gestantes porteuses des anticorps anti-VHC

	Effectif	Proportion
Manucure par du matériel commun	2	2/3
Intervention chirurgicale	2	2/3
Scarifications	2	2/3
Piercing	1	1/3
Transfusion sanguine	1	1/3
Tatouage	1	1/3
Multi partenariat sexuel	1	1/3
Consommation de drogue par injection	0	0/3

**Prévalence du diabète gestationnel chez les gestantes enquêtées:** parmi les gestantes enquêtées, 20 (7,9%) présentaient un diabète gestationnel. Parmi ces 20, aucune n´était porteuse des anticorps anti-VHC.

## Discussion

**Age:** la tranche d´âge la plus représentée dans la population d´étude était celle de 29,58 ± 5,5 ans avec des extrêmes de 14 ans et de 45 ans. La majorité était des femmes mariées (62,4%) et avaient une profession (79,8%). Les femmes porteuses des Ac anti-VHC dans l´étude avaient une moyenne d´âge élevée de 32 ± 3 ans avec des extrêmes de 30 et de 35 ans. Cette moyenne d´âge était plus élevée que la moyenne d´âge (de 29,58 ± 5,5) de l´ensemble des gestantes enquêtées. Ce résultat est similaire à celui de Ndong-Atome *et al.*[13] qui avaient trouvé une prévalence élevée chez les plus de 35 ans (p=0,05). Costa *et al*. [14] au Brésil en 2009 avaient trouvé que la tranche d´âge de gestantes la plus touchée par le VHC était celle supérieure ou égale à 40 ans. Au Cameroun, Richard Njouom *et al*. [15] avaient trouvé une prévalence élevée chez les femmes âgées de plus de 29 ans. Stoszek *et al*. [16] en Egypte avaient également signalé que l´âge élevé est un facteur de risque d´infection par le VHC chez la femme enceinte. En général, la prévalence de l´infection par le VHC est plus faible chez les femmes enceintes comparativement à la population générale, puisque les femmes en âge de procréer ont généralement moins de 40 ans [14]. Cela s´expliquerait par le fait que le VHC se transmet surtout par voie parentérale. Le vieillissement serait donc un risque connu d'infection par le VHC en raison de la longue période d´exposition au cours de la vie. Dans la présente étude, une seule des gestantes porteuses des anticorps anti-VHC connaissait le statut sérologique de son conjoint vis-à-vis du VHC qui serait séronégatif.

**Prévalence du portage des Ac anti-VHC:** chez les femmes enceintes, on estime la prévalence du VHC entre 0,15 et 8,6% au niveau mondial. Les taux les plus faibles se retrouvent encore une fois dans les régions les plus développées, comme l´Europe et les États-Unis (0,15 à 2,4%) et les plus hautes prévalences sont retrouvées dans des pays comme l´Égypte (8,6%) [17]. De manière globale la prévalence de l´hépatite C chez les femmes enceintes en Afrique est estimée à 3,4% [18]. Les plus faibles fréquences ont été retrouvées par Rouet *et al*. [19] en Côte d´Ivoire en 2004 (1%), Njouom *et al*. [15] (1,9%) chez des femmes enceintes au Cameroun en 2001. Par contre d´autres auteurs ont trouvé des proportions plus élevées : Kabinda *et al*. [20] en République Démocratique du Congo en 2013 (4,1%), De Paschale *et al*. [12] en 2014 à Tanguiéta dans le Nord Benin (7,4%), Ephraim *et al*. [21] au Ghana en 2013 (7,7%) et Stoszek *et al*. [16] en 2005 en Egypte (15,8%). Ce taux confirme la forte endémicité du VHC en Egypte qui détient la plus forte prévalence du VHC au monde [5]. La prévalence du portage des anticorps anti-VHC chez les femmes enceintes dans notre étude était de 1,2%. Develoux *et al*. [22] en 1991 avaient trouvé une fréquence de l´infection par le virus de l´hépatite C chez les gestantes à Cotonou au Bénin de 0,7%. Kodjoh *et al*. [23] avaient trouvé que le Nord Benin est une zone de forte endémicité pour les hépatites virales comparativement au Sud. Les variations entre les prévalences pourraient être en rapport avec des différences de la taille des échantillons, des tests utilisés et le fait que les études ne soient pas faites dans les mêmes groupes de populations. Selon une étude réalisée en 2018 par Bigna *et al*. [8], la prévalence de l´infection par le VHC chez les femmes enceintes était la plus élevée en Afrique du Nord (4,6%) et en Afrique de l´Ouest (3,3%). En Afrique centrale cette prévalence était estimée à 2,8% et en Afrique de l´Est à 2,1%. Notons que dans notre étude, une seule gestante se connaissait séropositive au VHC. Nous n´avons pas trouvé de co-infection VHB -VHC, ni de co-infection VIH-VHC. La prévalence du VIH dans la population étudiée était de 1,7%.

**Facteurs de risque:** de nos jours, l´instauration d´un programme de dépistage systématique des produits sanguins et l´amélioration des procédures de stérilisation a permis de réduire énormément la propagation du VHC par ces voies. La consommation de drogue par voie intraveineuse, les antécédents de tatouage, de piercings, les antécédents de soins hospitaliers «lourds» (dialyse, transplantation,..), les personnes exposées au sang, le fait d´avoir un proche porteur du VHC, l´utilisation de matériel médical recyclable (principal mode de contamination en Egypte) sont devenus la principale voie d´acquisition du VHC. Il faut ajouter en Afrique certaines pratiques traditionnelles telles que les scarifications ethniques, l´excision [4]. Dans notre étude, en ce qui concerne les facteurs de risque potentiels d´infection par le VHC, 1/3 avait un antécédent de tatouage et de piercing, 2/3 portaient des scarifications, 2/3 faisaient la manucure avec du matériel commun. Notons également que 2/3 avaient subi une intervention chirurgicale. On retrouvait une notion de transfusion sanguine chez une des gestantes (1/3). Kpossou *et al*. [24] en 2019, de même que Kodjoh *et al*. [23] en 2012 ont trouvé que les scarifications joueraient un rôle important dans la transmission des virus d´hépatites en l´occurrence le VHC au Bénin. Il en est de même pour Ndong-Atome *et al*. [13] qui ont rapporté que les procédures rituelles telles que les tatouages et les scarifications faciliteraient la transmission du VHC au Gabon. Ephraim *et al*. [21] au Ghana avaient trouvé que la transfusion sanguine, le tatouage, et le partage d´aiguille étaient associés au portage des anticorps anti-VHC. En Egypte, Stoszek *et al*. [16] ont identifié également les antécédents de transfusion sanguine comme facteur de risque de portage du VHC. Par contre Zenebe *et al*. [25] en Ethiopie n´ont identifié aucun facteur de risque dans leur étude. Il en est de même pour Elsheikh *et al*. [26] au Soudan.

Chez la femme enceinte, plusieurs études prospectives ont montré un taux de transmission materno-infantile du VHC au cours de la grossesse entre 5 et 7% en cas d´infection par le VHC seul, et entre 15 et 18% en cas de co-infection VIH-VHC [27]. La transmission peut se produire en intra-partum, per-partum, post partum. Le moment du plus grand risque est pendant l´accouchement du fait des contractions utérines qui rompent la barrière placentaire. Seules les mères virémiques dont la recherche d´ARN viral est positive sont capables de transmettre l´infection à leurs nouveau-nés [28-30]. La virémie maternelle et la co-infection avec le VIH sont les principaux facteurs associés à la transmission virale néanmoins quelques autres facteurs ont été relevés notamment des facteurs liés au VHC, des facteurs maternels, des facteurs liés à la grossesse et des facteurs immunogénétiques [28, 30]. Dans 20% des cas, le mode de transmission reste inconnue [31]. Par ailleurs, dans notre étude, une des gestantes porteuses des anticorps anti-VHC avait un antécédent de fausses couches spontanées. Karampatou *et al*. [32] ont trouvé que les femmes porteuses des anticorps anti-VHC avaient un risque élevé de fausses couches spontanées comparé à celles porteuses du VHB. Par contre Giakoumelou *et al*. [33] ont trouvé que l´infection par l´hépatite C ne semble pas avoir d´incidence sur l´issue de la grossesse. Il en est de même pour Floreani [34]. La plupart des études sur l´influence de l´hépatite C sur la grossesse ont donné des résultats peu concluants. Certains auteurs signalent très peu d´influence du VHC sur le développement de la grossesse et de l´accouchement [35-37]. Certaines études suggèrent un risque accru de diabète gestationnel chez les femmes enceintes porteuses des anti-VHC en raison de l´insulinorésistance engendrée par le VHC [12, 14, 38]. Toutefois aucune des trois gestantes porteuses des anticorps anti-VHC dans notre étude ne présentait un diabète gestationnel. Buresi *et al*. [39] en Colombie n´ont trouvé aucune différence entre la prévalence du diabète gestationnel chez des gestantes infectées par le VHC et celle dépistée dans la population générale. Les avis sont partagés sur le lien entre le diabète gestationnel et l´infection par le VHC.

## Conclusion

La prévalence des anticorps anti-VHC chez les femmes enceintes à Cotonou est faible (1,2%). Les facteurs de risque potentiels d´infection par le VHC retrouvés étaient : les scarifications, un antécédent d´intervention chirurgicale, un antécédent de transfusion sanguine, le piercing, le tatouage et la manucure avec du matériel commun. Une autre étude sur une cohorte plus importante est nécessaire pour mieux évaluer les influences réciproques entre la grossesse et l´infection au VHC d´une part et le lien avec le diabète gestationnel d´autre part.

### Etat des connaissances sur le sujet

- L´infection par le virus de l´hépatite C est faible chez les gestantes à Cotonou au Bénin, 0,7% d´après une étude réalisée en 1991;
- Au Nord du Bénin, précisément à Tanguiéta, la prévalence des anticorps anti-VHC est élevée: 7,4% selon une étude réalisée en 2014;
- Le dépistage du VHC pendant la grossesse n´est pas systématique.

### Contribution de notre étude à la connaissance

- La prévalence des anticorps anti-VHC chez les femmes enceintes à Cotonou a augmenté, mais demeure faible: 1,2%; peu de gestantes connaissent leur statut sérologique vis-à-vis de l´hépatite C;
- Le dépistage anténatal du VHC pourrait trouver un intérêt en vue de la planification d´une prise en charge ultérieure vu les résultats satisfaisants qu´offrent les nouveaux traitements à base d´antiviraux d´action directe;
- Il existe des facteurs de risque de portage des anticorps anti-VHC tels les antécédents de tatouage et de piercing, de scarifications, de manucure avec du matériel commun, d´intervention chirurgicale et de transfusion sanguine.
